# Improving the Friction-Wear Properties and Wettability of Titanium Through Microstructural Changes Induced by Laser Surface Treatment

**DOI:** 10.3390/ma18235410

**Published:** 2025-11-30

**Authors:** Agnieszka Twardowska, Łukasz Ślusarczyk

**Affiliations:** 1 Institute of Technology, University of the National Education Commission, Podchorazych 2, 32-084 Krakow, Poland; 2 Department of Production Engineering, Faculty of Mechanical Engineering, Cracow University of Technology, Al. Jana Pawła II 37, 31-864 Krakow, Poland; lukasz.slusarczyk@pk.edu.pl

**Keywords:** Ti grade 1, laser surface treatment, microstructure, resistance to wear, CoF

## Abstract

In this study, a surface treatment of Ti grade 1 was carried out in air with the use of a Yb-fiber laser to increase the friction-wear properties tested in dry contact with α-Al_2_O_3_. The laser surface treated specimens clearly differ in their surface roughness and wettability, coefficient of friction and resistance to wear, compared to untreated specimens. The microstructure changes induced by laser treatment were investigated using confocal scanning electron microscopy with chemical composition analysis by energy-dispersive spectroscopy, and phase composition by X-ray spectroscopy. It was found that laser surface treatment caused the formation of titanium oxide layers with TiO_2_ (rutile, anatase and brookite) as the main constituent, while in the subsurface areas a partial transformation of α-Ti to β-Ti or α′-Ti was thermally induced. Specimens containing β-Ti or α′-Ti in the subsurface area and anatase or brookite in the top layer were characterized by two times lower friction coefficient values and 10 times lower volume wear index Wv in comparison to untreated Ti grade 1. Results clearly confirmed the beneficial effect of laser surface treatment on friction-wear properties of Ti grade 1, but the selection of laser processing parameters was crucial both for resistance to abrasive wear and wettability.

## 1. Introduction

High expectations towards products designed for aviation, aerospace or especially for medical applications, cause interest in lightweight materials with chemical compositions and properties that ensure not only high durability but also safety for the potential user [1,2,3]. Titanium and titanium alloys are an important group of materials developed for these applications due to their special properties, i.e., low density, favorable strength to yield strength ratio and good corrosion resistance [4,5]. The latter property is related to the high affinity of titanium to oxygen and the formation of a stable passive oxide surface layer, tightly adhering to the substrate and composed mainly of TiO_2_ (rutile), but also some amount of Ti_2_O_3_ and TiO underneath [6,7]. One of the important concerns about the use of titanium and its alloys is its low resistance to wear [8,9]. The introduction of alloy additives is an effective method of improving properties, but in the case of medical applications, some alloy additives pose risks to the health of patients. An example of such an alloy is Ti6Al4V, for which it has been found that the presence of Al and V additives cause allergies in patients [10], therefore research has been undertaken to develop titanium alloys without allergenic alloying elements [11] or to re-engage in the improvement of titanium technical purity. Ti grade 1 (UNS R50250/W. Nr 3.7025, Table 1) does not contain allergenic elements, either in the form of additives or impurities. Mechanical properties and resistance to wear of this grade are low in comparison to Ti alloys, because chemical purity excludes the mechanisms of solution hardening and dispersion-hardening, so the only commonly used mechanism for strengthening of Ti grade 1 is by cold working. Considering that the friction-wear properties are primarily related to the surface of the products, some attempts are also made to use surface treatment methods including those using laser beam [12,13]. The rapid growth of interest in laser treatment of materials, including those used in surface engineering is due to the development and availability of new laser sources, which is possible thanks to the acceptable price of the devices. Laser surface treatment, depending on the working mode (pulsed, continuous work) and parameters used, can cause significant changes in the microstructure of materials and thus also in their properties [14]. These changes can be the result of various phenomena that are possible when the laser beam interacts with matter, i.e., ablation, melting (and further amorphization or rapid crystallization), rapid heating and cooling afterwards, or only heating or excitation [15]. As a result of the above-mentioned phenomena, significant changes occur in the microstructure of the material: strong grain refinement, extension of the limit of solid solubility of additives, diffusionless (martensitic) phase transformations, formation of an unusual metastable phases or new ones. Laser surface treatment methods are energy-efficient and material-saving. They do not generate waste; therefore, they are recognized as environmentally friendly and zero-emission. In the literature, attempts are described to use laser energy to modify titanium, but they mainly focused on Ti alloys, in particular on Ti6Al4V [12,14,16]. The laser surface treatment seems to be not applied to Ti grade 1, although this particular grade is an important biomaterial, widely used for dental implants, anastomosis plates, and screws in maxillofacial surgery [3]. High purity of Ti grade 1 ensures a low risk of implant rejection or allergic reactions in potential patients, but the price paid for high biotolerance is low mechanical properties and resistance to wear. The latter is believed to be the cause of loosening of dental implants, screws, and mounting plates in bone tissue [1,2,3]. Surface treatment methods can effectively increase the durability and stability of the connection by microtexturing the implant surface and/or creating layers that facilitate integration with the surrounding tissue. Alumina, hydroxyapatite and titania thin films as well as porosity formed in the surface layer on titanium promote osseointegration of the titanium implants [3,17]. Surface microtexturing is also helpful and easily achieved, particularly through laser processing. It has been shown that achieving the desired surface roughness through laser processing allows for changes in surface wettability, making it hydrophilic or hydrophobic, depending on the needs [18].

Based on the state of knowledge regarding laser processing of high-purity commercial titanium for medical applications presented above, we have identified gaps in this research area. Therefore, in this study, we attempted to fill this gap and selected Ti grade 1 as the subject of research to determine whether laser surface processing performed in an ambient atmosphere is effective in increasing the wear resistance of Ti grade 1. To achieve this goal, we conducted a series of studies and tests, including the microstructure examination (including chemical and phase composition, topography analysis with surface roughness measurement), friction-wear properties, and surface wettability of grade 1 titanium before and after laser treatment with Ringer’s solution. The resistance to wear and coefficient of friction in a ball-on-disk geometry was studied under dry friction conditions, using α-Al_2_O_3_ as a counterpart material. This is the first in-depth study of the wear resistance of Ti grade 1 after surface laser treatment with ytterbium-fiber laser. No research was found regarding the effect of such treatment on the friction-wear properties of this titanium grade and the originality of the study lies in the determination of the friction-wear properties of Ti grade 1 after surface laser treatment in the friction dry contact with alumina which is used as a bone replacement material.

## 2. Experimental

### 2.1. Materials

The material was the Ti grade 1 (Hempel Special Metals, Sosnowiec, Poland) in the form of 16 mm × 16 mm plates cut out from a 4 mm-thick sheet using a corundum disk and precision disk cutter with a closed water-cooling system. To remove the surface layer of the titanium sheet created in the rolling process, the specimens were ground and polished on one side. Grinding was carried out on SiC abrasive papers with #200–#2000 grit size. Then, the process of polishing was carried out. For this purpose, MD Struers disks (MD-Dac, MD-Chem, Copenhagen, Denmark) and alumina suspensions (AP-A, Struers) with grain sizes of 3 and 1 µm were used; finally polishing was carried out with silica suspension (OP-S, Struers 0.25 µm). Between the individual steps, the specimens were rinsed in water, then in an ultrasonic cleaner in a water bath, degreased in pure isopropanol, and dried with warm air.

For the microstructure examination by electron scanning microscopy (SEM), cross-sections of laser-treated specimens were prepared in the same way and additionally were etched in Kroll’s reagent (2 mL HF, 2 mL HNO_3_, 100 mL water).

### 2.2. Laser Surface Treatment Parameters

In the process of laser treatment of the surface of titanium grade 1 specimens, the pulsed ytterbium-doped fiber laser was used (60W MOPA, Raycus, Wuhan, China) operating at λ = 1064 nm. Laser processing surface treatment was carried out in air, at room temperature, with the following parameters: power density 6 × 10^8^ W/cm^2^, spot diameter 0.05 mm, line distance 0.1 mm, repetition frequency—10, 20 and 48 kHz, pulse length 100 ns, scan speed 80 mm/s, line by line, one pass, ~10% overlapping. The parameters of the laser surface treatment were selected using the trial and error method. We conducted a number of trials of laser surface treatment of Ti grade 1 with variable parameters, i.e., power density 1.5 × 10^7^ W/cm^2^–1.5 × 10^9^ W/cm^2^, repetition frequencies 10 kHz, 20 kHz and 48 kHz, pulse length 100 ns, and then surface topography observations were carried out by confocal microscopy with surface roughness measurements conducted on recorded 3D images. Based on the results of these preliminary studies, 3 variants of parameters were selected for the most promising surfaces, i.e., those created as a result of heat treatment with the smallest possible melting and maintaining high surface smoothness.

### 2.3. Surface Topography Observation and Roughness Measurement

Laser-treated specimens were subjected to detailed microstructural examination and roughness analysis by confocal microscope Olympus LEXT™ OLS5100 3D (Olympus, Tokyo, Japan) operating at laser wavelength λ = 410 nm, with integrated software for the analysis of surface topography and roughness parameters measurement (S_a_—arithmetical mean height, S_z_—maximum height) on the basis of recorded 3D images.

### 2.4. Microstructure, Chemical and Phase Composition Examinations

Detailed microstructural studies were carried out before and after laser treatment as well as after the friction-wear test, using scanning electron microscopy SEM (JEOL JSM 6610 LV, JEOL Ltd., Tokyo, Japan). Chemical composition was examined in micro areas by energy dispersive spectroscopy (EDS) which accompanied SEM observations. The phase composition of Ti-grade 1 and its changes as a result of laser surface treatment were carried out by means of X-ray diffraction spectroscopy in Bragg–Brentano geometry using MiniFlex2 X-ray spectrometer by Rigaku Corporation, Tokyo, Japan, (Cu_Kα1_, λ = 1.54 Å, V = 30 kV, I = 15 mA, step size 0.02°, scan speed 1°/min). For phase identification, a database and PDXL 2.9 Integrated Powder X-ray Diffraction Software by Rigaku were used.

### 2.5. Friction-Wear Test Parameters

Friction-wear tests were conducted in accordance with ASTM G99-05 [19] in ball-on-disk configuration, i.e., with a stationary ball made of a hard material (α-Al_2_O_3_, 3.175 mm in diameter, hardness ~15 GPa) pressed with 1 N against the surface of a rotating tested specimen. The sliding distance was 201.13 m, testing time 2000 s, rotation speed 0.192 m/s, rotation diameter 10 mm, humidity 30%, temperature 22 °C.

### 2.6. Wettability Tests

Wettability tests were carried out using Ringer’s physiological solution (8.6 g/L NaCl, 0.3 g/L KCl, 0.48 g/L CaCl_2_), freshly prepared in our laboratory, using high purity chemicals and deionized water. The droplet (10 µL) was applied using an automatic micropipette. The measurement was carried out at 22 °C in air (35% humidity); two trials were performed for each specimen, in various areas of the tested surface. The contact angle of the droplet deposited on the surface was measured based on the images recorded with a digital microscope.

## 3. Results and Discussion

**Figure 1:**
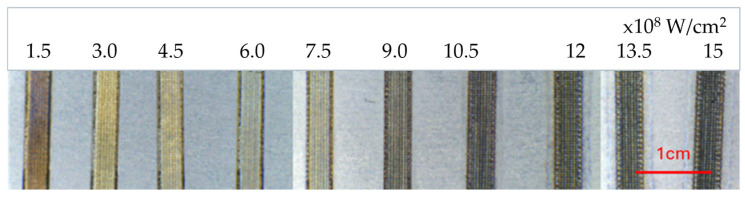
Titanium grade 1 test specimens after laser surface treatment at variable power density (1.5 × 10^8^ W/cm^2^ to 1.5 × 10^9^ W/cm^2^, pulse repetition frequency 48 kHz).

The parameters of laser surface treatment were selected based on preliminary experiments, conducted with variable processing parameters, i.e., energy density (fluence), pulse width, frequency of repetitions and speed of scanning. These parameters directly influence the amount of energy delivered to the material. Figure 1 shows examples of the titanium grade 1 test specimens after laser surface treatment at variable power densities, i.e., 1.5 × 10^8^ W/cm^2^ to 1.5 × 10^9^ W/cm^2^.

### 3.1. Surface Roughness Before and After Laser Treatment

**Figure 2:**
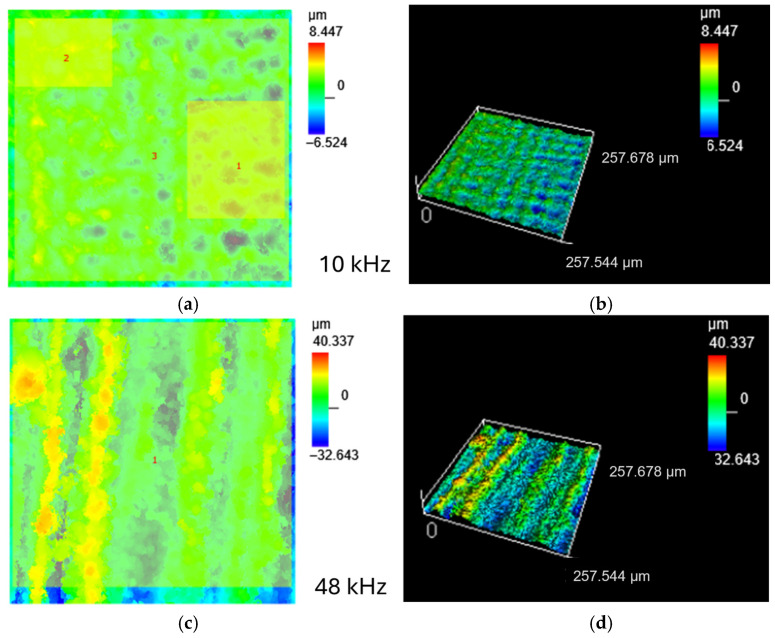
Titanium grade 1 after laser surface treatment at 10 kHz and 48 kHz: (**a**,**c**) 2D images (in height) with marked areas (in yellow) for surface roughness measurement S_a_ and S_z_, (**b**,**d**) 3D images registered by confocal microscopy.

A detailed analysis of the surface topography of titanium grade 1 after grinding and polishing and after subsequent laser surface treatment was carried out using a confocal microscope CM, enabling the measurement of roughness parameters based on recorded 3D images. Figure 2a,c show examples of registered 2D maps (height) with marked areas used for surface roughness measurement. Figure 2b,d are 3D images used for calculation of S_a_ and S_z_ surface roughness parameters. For each specimen, measurements were made in three different areas; a summary of the results is presented in Table 2.

### 3.2. Microstructural Changes, Surface Roughness of Laser Treated Specimens

The specimen treated with the highest repetition frequency, i.e., 48 kHz, was characterized by the highest roughness: S_a_ = 10.1 ± 1.5 μm and S_z_ = 65.4 ± 6 μm. The smoothest surface was obtained for the specimen processed at the pulse repetition frequency of 10 kHz; both average values of roughness parameters were lower than those determined for the reference specimen. Their differences fall within measurement uncertainty. Higher values of roughness obtained for the reference specimen result from the way in which S_a_ and S_z_ parameters are calculated. S_a_ is calculated by summing the absolute differences between each point’s height and the average height over the entire surface, then dividing by the surface area. Some relatively deep scratches which were formed during grinding and not fully removed by subsequent rough polishing strongly affected the values of surface roughness because the surface roughness parameters for mirror-polished titanium are at the level of several nanometers; however, this requires considerable care and often the use of chemical or electrochemical polishing. Nevertheless, the surface roughness values before and after laser treatment at 10 kHz and 20 kHz allow us to conclude that with properly selected laser treatment parameters it is possible to improve the surface quality (by removing or shallowing scratches) and its uniformity.

**Figure 3:**
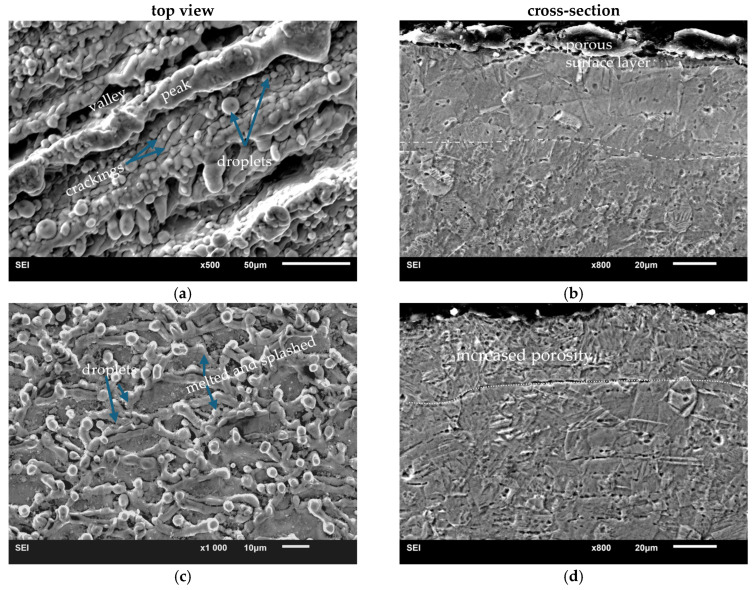

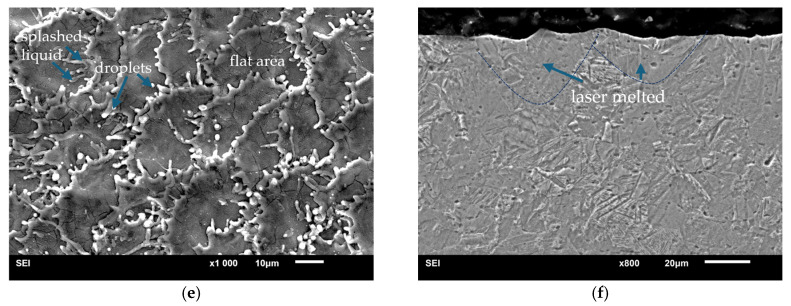
Secondary electron images SEI of the microstructure of Ti grade 1 after laser surface treatment: (**a**,**b**) 48 kHz; (**c**,**d**) 20 kHz; (**e**,**f**) 10 kHz.

A much broader insight into the influence of laser processing parameters on the surface quality was provided by observations of surface morphology and cross-sections using scanning electron microscopy methods (Figure 3). The most developed surface was obtained after laser processing at 48 kHz (Figure 3a), on which clear peak and valley topography was observed. The surface was covered by spherical particles with diameter < 10 μm. Most of the particles are isolated, some, especially the smaller ones, merge. Spherical-shaped particles observed after laser processing of metals are droplets formed as a result of local melting [15]. Droplets are also visible on the surfaces of two other specimens, but they are less numerous, and in the case of laser processing at 10 kHz, spherical droplets are few (Figure 3f). The thickness of the subsurface layer changed as a result of laser treatment can be determined from the position of the pore line formed at a certain depth from the sample surface (marked with a white dashed line), Figure 3b,d. In the case of laser processing at 10 kHz, this pore line does not appear. The presence of melted areas is evidenced by rounded boundaries of subsequent laser spots (Figure 3e,f).

For specimens processed at 20 kHz and 10 kHz, slightly overlapping spots formed a distinct pattern, especially evident in the case of a specimen treated at 10 kHz. The estimated depth of a single spot (marked with blue line, Figure 3f) is approx. 30 μm. Inside the spot the surface is almost smooth. On the other hand, the solidified ‘splashed’ edges of the spots testify to the dynamics of the solidification process. Overheating under dynamic laser action caused liquid flow and splashing, accompanied by evaporation or ablation [15]. The latter is responsible for the porosity of the top layers visible on the cross-sections (Figure 3b,d,f). Another cause of the porosity formation is high solubility of oxygen in titanium, which, under rapid heating and subsequent cooling after laser treatment, promotes the entrapment of absorbed gas in the solid during crystallization.

### 3.3. Changes in Phase Composition

**Figure 4:**
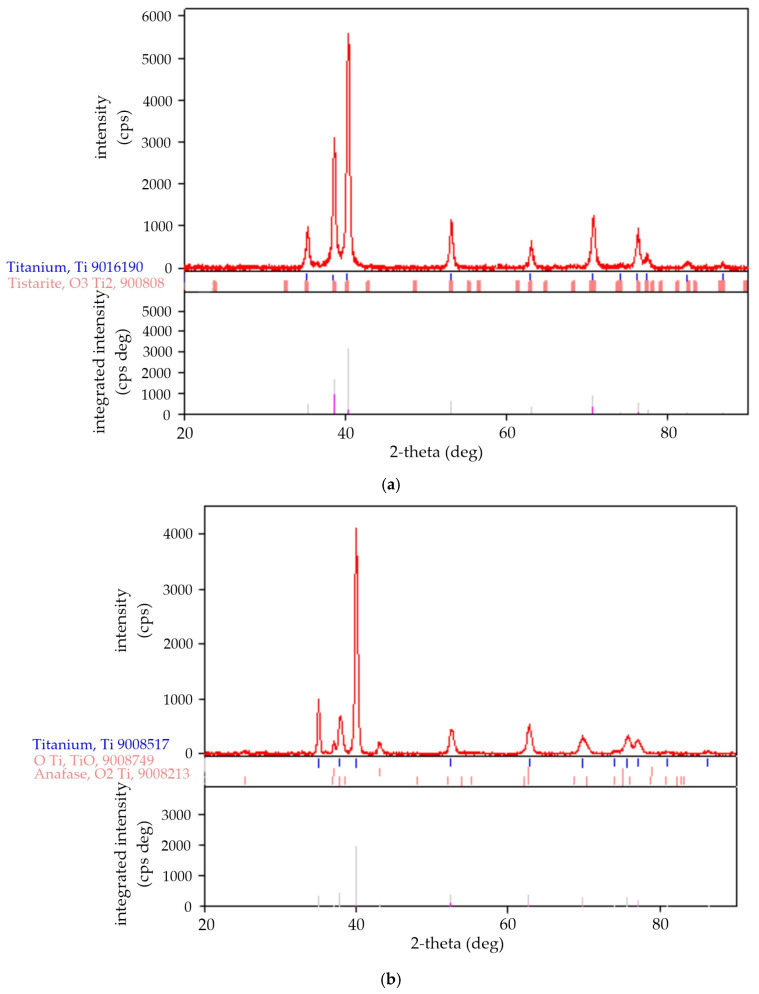

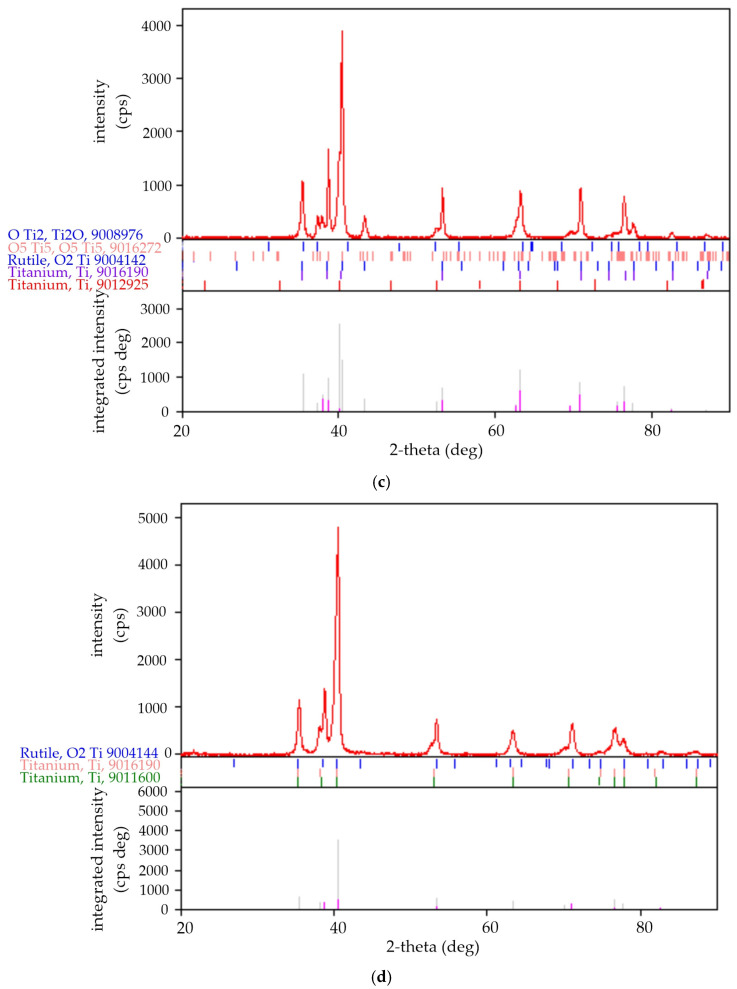
XRD spectra registered for Ti grade 1 (**a**) before and after laser surface treatment at (**b**) 48 kHz, (**c**) 20 kHz, (**d**) 10 kHz. Below the diffraction patterns, the positions of maxima of the standard used for phase identification are given with the integrated intensities calculated for the main identified phases (α-Ti—gray, titanium oxide—pink).

Figure 4 shows the XRD spectra recorded for Ti grade 1 before and after the laser surface treatment. Comparing the XRD spectra recorded for titanium grade 1 (Figure 4a) and laser surface-treated (Figure 4b–d), some differences were found in the relative intensity of the maxima but also in their number: the presence of additional maxima for laser-treated specimens that were not present in the XRD spectrum of untreated titanium. A detailed analysis of XRD spectra was carried out using Rigaku software and PDF standards database, which allowed us to identify the phases occurring in the studied specimens and determine their quantity using the Rietveld method. Based on the results, it was found that laser treatment of the titanium surface carried out in air leads to phase transformations of titanium and the formation of titanium oxides of different Ti-to-O ratios. These changes concern both the stoichiometry of the resulting titanium oxides and their crystalline form, as presented in Table 3.

The main phase component of untreated titanium grade 1 is α-Ti (hex. space group 194: P63/mmc). The presence of this phase was found in each of laser surface treated specimen as the dominant one, which is understandable due to the surface nature of laser treatment. In the case of the specimens treated at 20 kHz and 10 kHz, in addition to the alpha phase, β-Ti phase was identified (20 kHz), bcc 194: P63/mmc (2.936 Å, 2.936 Å, c 4.663 Å) and martensitic phase α′-Ti (10 kHz), hex. 191: P6/mmm (4.493 Å, 4.493 Å, 2.754 Å). The martensitic transformation in titanium occurs at high cooling rates from temperatures above the martensite start temperature, where the bcc phase transforms into hcp in a diffusionless transformation process. In addition to the martensitic hexagonal α′-Ti phase, an orthorhombic phase α″-Ti can be formed, the latter being observed during quenching from temperatures below approximately 900 °C [5]. This phase was not identified in our specimens, so the diffusionless transformation temperature in specimen treated in 10 kHz was higher than 900 °C. The total content of Ti phases in the specimen treated at 10 kHz was 67%, which is the lowest value among the analyzed specimens. Therefore, it can be concluded that the reduction in the repetition frequency causes a decrease in the content of titanium phases in total in favor of titanium oxides. Lower repetition frequencies promote a partial conversion of the α-Ti phase into the beta phase (20 kHz) or to martensitic phase α′-Ti (10 kHz). The degree of transformation increases with decreasing repetition frequency and in the specimen treated at 10 kHz, the content of α-Ti is only 40%. Martensite transformation in titanium occurs upon quenching from the high temperature β-phase field [17]. In our case, this transformation happened during the laser treatment at 10 kHz and it was accompanied by an increase in calculated density, exactly as the literature sources state [15], while in the specimen treated at 20 kHz the martensite transformation was not evident or did not occur, and as a result, an α/β-Ti microstructure was formed in the subsurface layer. Such changes in phase composition of titanium and its alloys are often observed after the laser treatment [12,16,20]. In addition to titanium (α, β and α′-Ti), the presence of titanium oxides was identified in all specimens (Table 3).

### 3.4. Resistance to Wear and Coefficient of Friction

**Figure 5:**
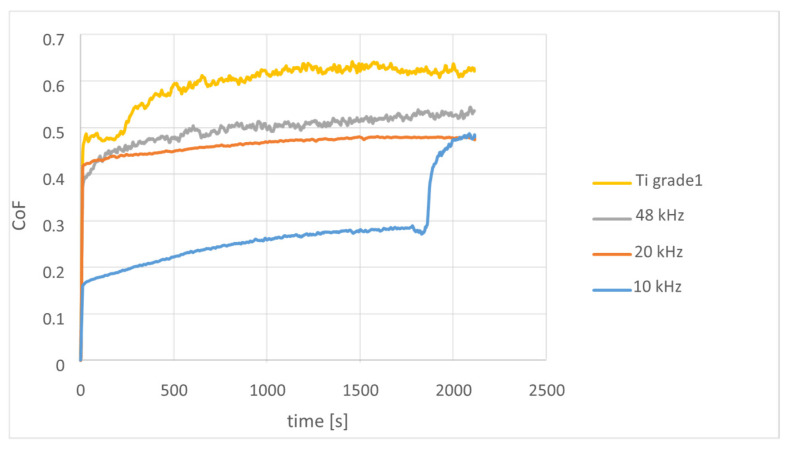
Changes in the friction coefficient CoF in the dry friction contact with α-Al_2_O_3_ counter-specimen for Ti grade 1, before and after laser treatment.

The changes in the Wv index and the coefficient of friction determined in the dry contact with corundum are shown in Figure 5 as a function of the duration of the friction-wear test. In the analyzed combination of friction-wear pairs, each of the applied variants of surface laser treatments resulted in a decrease in the friction coefficient. The lower the frequency of laser repetition used, the lower the value of the CoF averages, calculated for individual specimens over the entire ball-on-disk test run (Table 4).

In the case of a non-treated specimen, the CoF value initially stabilizes at the level of 0.5 up to about 250 s, which probably is related to the existence of a thin oxide layer that was formed naturally on the titanium surface, which according to the results of the XRD analysis presented previously was identified as Ti_2_O_3_. However, after 250 s, this oxide layer is removed and further interaction with the corundum ball takes place in worse conditions, as indicated by the rapid increase in CoF to 0.6 (reached at 500 s), then to 0.62 and stabilization at this level. The CoF vs. test time plot is characterized by significant fluctuations in CoF values, similar to the graph recorded for the specimen treated at 48 kHz, which is surprising, considering the surface roughness of the untreated specimen (ground and polished) while the latter one was the roughest of all the tested specimens. Thus, the reason for the fluctuation of the CoF value is not directly linked to the surface roughness of the tested specimen and should be linked to other factors, e.g., the type of titanium oxide present on the surface or formed as a result of tribo-chemical reactions of titanium with oxygen (Magnéli phases) and their low friction or lubricating properties.

The other two graphs (for 20 kHz and 10 kHz) are characterized by significantly smaller fluctuations of the measured CoF values, which indicates a much more stable interaction between the ball and the specimen. For a specimen treated at 20 kHz, the coefficient of friction increases almost linearly from ~0.42 to ~0.48, while the CoF vs. test time plot registered for a specimen treated at 10 kHz is more complex. In the range of up to ~1800 s, the CoF value slowly increased from ~0.19 to ~0.28, then rapidly increased in less than 200 s to ~0.48 and stabilized at this value. The mean CoF value calculated for this variant was 0.24 and is the lowest value calculated for all tested specimens. On the basis of the plot registered for specimen treated at 10 kHz shown in Figure 2, it can be concluded that in the range up to 0.3 CoF the value was caused by the presence of a titanium oxide layer, which after this time was worn and further interaction with the corundum ball took place in less favorable conditions, similar to those that occurred in the case of the specimen laser-treated at 20 kHz, because even after removing this surface layer, the coefficient of friction is still lower than that measured for the titanium specimen which was not subjected to laser surface treatment. CoF stabilization at the same level as for the 20 kHz specimen suggests that when the surface layer produced by laser treatment at 10 kHz was removed, there were still changes in the material in the subsurface area like those obtained for the 20 kHz layers. Compared to the oxide layer on untreated titanium, the oxide layer on the specimen treated at 10 kHz is much more durable, which may be due to its greater thickness and/or more favorable phase composition and/or stronger bonding to the subsurface layers. According to the results of the XRD analysis, in the specimen treated at 10 kHz, titanium oxides are constituting 33% wt. of the analyzed material, of which 29% wt. are Ti_2_O and Ti_5_O_5_. Both oxides are formed under oxygen deficiency, as discussed earlier. Therefore, it can be concluded that laser treatment of the surface of Ti grade 1 carried out in air causes a decrease in the coefficient of friction in the friction combination with corundum, as a result of phase transformations in the subsurface area and the formation of titanium oxides on the surface. The type and quantity of titanium oxide is important and depends on the laser treatment parameters, but, in general, the presence of an oxide layer is beneficial for the CoF value. In the Ti-O system, there are many phases that differ in the Ti-to-O ratio, but the most stable among them are titanium dioxides with the molecular formula TiO_2_, which can occur in three allotropic forms (anatase, rutile and brookite) [21]. Rutile (tetragonal, P42/mnm) and anatase (tetragonal, I41/amd) are the most common. In our experiments they have also been identified in allotropic forms, but only in the laser-treated specimens. Anatase was identified only in a specimen processed at higher repetition frequency, i.e., at 48 kHz together with a small (7.9% wt.) content of TiO (cubic, 225: Fm-3m). Rutile occurred in specimens laser treated at lower repetition frequency (20 kHz and 10 kHz). The content of rutile was 23% in the specimen treated at 20 kHz and only 4%—for 10 kHz. In the latter case, higher contents were achieved by oxides with a higher Ti-to-O ratio than 1:2, i.e., Ti_2_O (hex, 191: P6/mmm) with a content of 15% wt. and Ti_5_O_5_ (monocl. 12:112/m, unique-c, cell-1)—14% wt. These oxides were not identified in other specimens. In the untreated reference specimen, the only identified titanium oxide was Tistarite Ti_2_O_3_ (Table 4). This oxide is a component of a naturally forming layer on the surface of titanium in contact with atmospheric oxygen under conditions of oxygen deficiency, which in our case is the result of a short exposure of the specimen surface to the air before performing the XRD measurement. According to the titanium-oxygen equilibrium diagram, the high-temperature oxidation conditions of titanium are created during the laser treatment, as a result of which the formation of TiO, Ti_2_O_3_, Ti_n_O_2n−1_ Magnelli phases and TiO_2_ rutile oxides was expected [22]. Studies of the oxidation process of titanium [23] have shown that the phase composition and morphology of the oxide layer formed on titanium depend on the temperature and time of oxidation. Up to a temperature of approx. 650 °C, rutile is the only oxidation product. As the temperature rises, next to rutile, other titanium oxides are also formed, among which Ti_2_O_3_ is predominantly present. At oxidation temperatures exceeding 800 °C, in addition to TiO_2_ and Ti_2_O_3_, the corrosion products also include TiO oxide and Magnelli Ti_n_O_2n−1_ [8]. Given that the titanium melting point (1668 °C) was exceeded during the laser treatment in air, the presence of these oxides should not be surprising.

The formation of metastable phases (crystalline and amorphous) is one of the most important advantages of laser processing of materials [15], not only in the laser surface treatment but also in laser welding [24], or coating deposition by the PLD method [25]. In the case of laser surface treatment of Ti grade 1, in addition to metastable Ti phases, there were also phases from the Ti-O system.

**Figure 6:**
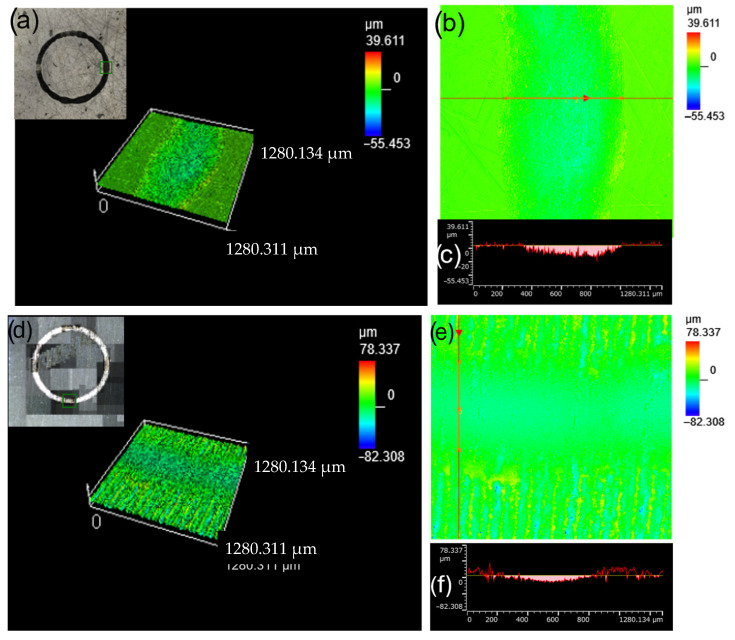
Measurement of volume loss (optically) based on a 3D images of the worn surface: (**a**,**d**) 3D images by confocal microscopy with general view of wear track (inserted); (**b**,**e**) 2D (height) maps with the profile measurement line; (**c**,**f**) profile line with marked area used for calculation of cross-sectional area of the friction path; (**a**–**c**) Ti grade 1 (untreated); (**d**–**f**) laser-treated at 48 kHz.

The values of the W_v_ index were calculated using two methods. Figure 6 shows two examples of volume loss measurement based on 3D images of the worn surface (Figure 6a,d) for untreated Ti grade 1 (Figure 6a–c) and the laser-treated specimen (Figure 6d–f), with wear profiles and examples of cross-sectional area calculations that were used to determine the volume loss W_v_ (op) according to Equation (1).

(1) $$W_{v}=\frac{V_{L}}{F_{n}\times L}$$

where

V_L_—volume of worn material [m^3^];

F_n_—load applied [N];

L—sliding distance [m].

In our case, two methods of determining the volume loss V_L_ were used:

- V_Lo_—the volume of worn material calculated on the basis of measurement of the cross-sectional area of the friction path using an optical profilometer (confocal microscope), according to ISO 8295:1994 [26];
- V_Lm_—the volume loss calculated by a difference in weight of the tested specimen before and after the friction-wear test, with respect to the theoretical density of the tested specimen, according to Equation (2).

The measurement of volume loss using the optical method had limitations resulting from the very small depth of the wear track profile and the value of the measurement error of the cross-sectional area in the case of the specimens treated at 10 kHz and 20 kHz (the same values were obtained). Since we have already had such a case, before and after the test we weighed the specimens to be able to determine volume loss by mass difference W_v_ (m), according to Equation (2).

(2) $$W_{v}=\frac{m_{a}-m_{b}}{F_{n}\times d\times L}$$

where

W_v_—wear index;

d—theoretical density of tested specimen [g/cm^3^];

m_a_, m_b_—weight of the specimen (m_a_—after the test, m_b_—before the test) [g];

F_n_—normal force [N];

L—sliding distance [m].

The results presented in Table 4 confirm that all the selected parameters of laser treatment allowed an increase in the wear resistance of Ti grade 1. The laser treatment parameters are of key importance for the improvement of the resistance to wear by friction. In our experiment, an increase in wear resistance of at least ten times was achieved, which shows the potential of using laser surface treatment of Ti grade 1.

**Figure 7:**
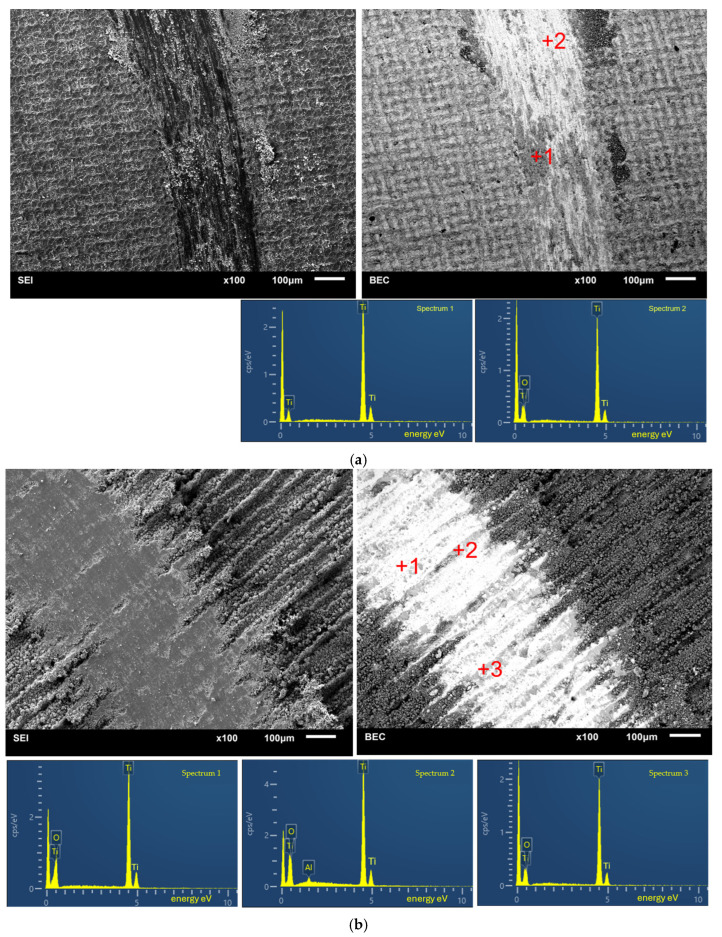
SEM images of wear track in laser-treated Ti grade 1: (**a**) 10 kHz, (**b**) 48 kHz with EDS spectra taken from marked areas.

Figure 7 shows SEM images of the laser-treated surfaces of Ti grade 1 after a friction-wear test. There are clear differences in chemical composition in the wear track (spectra 1 and 2, Figure 7a) with respect to O. The SEM image shows the wear products on the wear edge and inside it. Particles come from a layer of oxides produced during the treatment, while there is a Ti substrate underneath. For the specimen treated at 48 kHz (Figure 7b, spectra 1–3), the traces of Al are also visible as the effect of alumina rubbing into the wear track. The specimen had relatively high surface roughness with a peak and valley morphology, which were smoothed out in the frictional path, enriching themselves in Al and additionally with the O content from α-Al_2_O_3_ ball.

### 3.5. Effect of Laser Processing on the Wettability of Ti Grade 1 Surfaces

**Figure 8:**
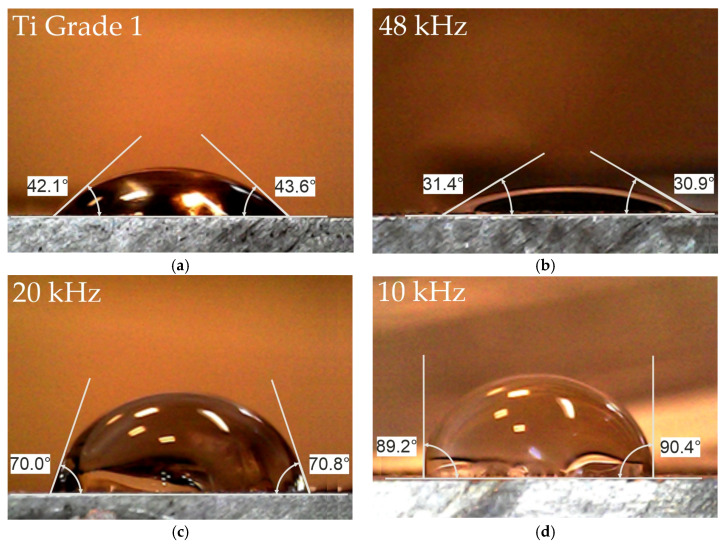
Effect of laser treatment on the apparent contact angle of Ringer’s solution for Ti grade 1: (**a**) untreated, (**b**–**d**) after laser surface treatment.

Laser treatment has clearly affected the surface wettability of Ti grade 1 specimens. Depending on the parameters used, the laser treatment allowed for increasing or decreasing the wettability of the surface. In the initial state (after grinding, polishing and degreasing), the surface is well wetted by the Ringer’s solution, the contact angle is ~43° (Figure 8a). The superhydrophilic surface with the lowest contact angle of ~31° was obtained by the laser treatment at 48 kHz. For the other two variants, the contact angle is higher: ~70° for the one treated at 20 kHz and ~90° for 10 kHz (Figure 8c,d). Since the roughness parameters S_z_ and S_a_ (Table 2) for the surface laser treated at 10 kHz are similar to the roughness of the untreated Ti grade 1 specimen, the key to the wettability of is not the roughness itself, but the type of oxide layer present. In the case of surfaces treated at 10 kHz, rutile TiO_2_ was the main titanium oxide constituent, while in the untreated Ti grade 1 the only identified titanium oxide was Tistarite Ti_2_O_3_. Changes in the contact angle of titanium after laser texturing in air were studied in detail as a function of energy density with respect to roughness and chemical bonds investigated by Raman microspectroscopy [27]. They demonstrated that wettability of a titanium surface by water, depends not only on the topography but also on the presence of oxides, including Ti_3_O_5_, anatase, and/or rutile TiO_2_ formed on the surface after laser irradiation. Unfortunately, it has not been shown how the presence of individual oxides affects the wettability of the surface, but it is generally assumed that the wettability of a substrate by a fluid is determined by the surface’s topography and the surface’s physical and chemical properties [28]. According to [29], rutile is the worst hydrophilic form of titania, with highest contact angle of ~73.9°, which is consistent with the value of the contact angle we obtained for the surface treated at 20 kHz, in which the only titanium oxide is indeed rutile. The presence of rutile could therefore have had a negative effect on the wettability of the surface treated at 20 kHz. But its wettability is better than that of the surface treated at 10 kHz; therefore, we believe that the reduced wettability of the surface treated at 10 kHz is related not only to the presence of rutile, but also to the other two identified titanium oxides, i.e., Ti_2_O and Ti_5_O_5_, which give the surface a much more hydrophobic character.

## 4. Conclusions

The paper presents the results of the study aimed at increasing the wear resistance of Ti grade 1 by surface treatment with the use of a pulsed laser beam. Based on the study conducted in the field and the results obtained, the following was found:

- Laser surface treatment is an effective and environmentally friendly method of increasing friction-wear properties of titanium grade 1 with respect to resistance to wear and coefficient of friction in friction contact with alumina.
- Laser treatment of Ti grade 1 carried out in air caused significant changes in the phase composition of the treated material, both in the resulting surface layer and in the subsurface area.
- In the surface layer of Ti grade 1, which is formed as a result of laser treatment in air, titanium oxides with different Ti-to-O ratios are formed. The main oxide is TiO_2_, which, depending on the processing parameters, can occur in the form of rutile, anatase and/or brookite in various contents.
- In the subsurface areas (under the titanium oxide layer) laser treatment causes partial phase transformation of α-Ti to β-Ti or to the martensite α′-Ti phase.
- The changes in the microstructure of Ti grade 1 in the surface layer formed during the laser treatment in air and in the subsurface heat affected zone are of key importance for the achieved improvement of friction-wear properties.
- Laser treatment of titanium surfaces carried out in air allows the wettability control in a wide range of contact angles. It was demonstrated that it is possible to obtain a superhydrophilic or hydrophobic surface, depending on the type of the surface pattern, roughness, and the phase composition of the oxides formed on it.
- Among the selected variants of laser treatment parameters of Ti grade 1, the most advantageous in terms of superphilicity is the 48 kHz variant, but it increases the surface roughness significantly.

The most advantageous, due to the smoothness of the surface, wear resistance, and low CoF value is the 10 kHz variant. Further work to optimize the laser surface treatment process of this grade of titanium is underway.

## Figures and Tables

**Table 1:** Chemical composition and selected properties of Ti grade 1 (UNS R50250/W. Nr 3.7025).

Chemical Composition [% wt.]						Tensile Strength TSt [MPa]	Yield Strength YS [MPa]	Hardness HRC/HRB	Young Modulus E [GPa]	Density d [g/cm^3^]
Ti	Fe	O	C	N	H					
99.5	0.20	0.18	0.080	0.030	0.015	240	172	70/120	105	4.51

**Table 2:** Roughness parameters measured optically by CM for Titanium grade 1 after mechanical grinding and polishing and after subsequent laser surface treatment at different repetition frequencies.

Specimen	S_z_ [µm]	S_a_ [µm]	Surface Features
Ti grade 1	20.4 ± 2	5.1 ± 0.2	Scratches of different depths and directions left after grinding
48 kHz	65.4 ± 6	10.1 ± 1.5	Strongly developed ‘peak and valley’ surface, droplets and pores
20 kHz	44.1 ± 5	2.4 ± 0.5	3D patterned surface, ripples and splashes
10 kHz	18.1 ± 1	1.2 ± 0.02	Homogeneously distributed cavities (pits) after ablation with minor melting at the edges of the pathways

**Table 3:** Phase composition of Ti grade 1, before and after laser surface treatment.

Specimen	Phase Name	Content [% wt.]	Space Group	a, b, c [Å]	Volume [Å^3^]	Card Number	Calc. Density [g/cm^3^]
Ti grade 1 untreated	α-Ti	99.0	194: P63/mmc, hex.	2.95, 2.95, 4.679	35.265	9016190	4.508
	Tistarite Ti_2_O_3_	1.0	167: R-3c, hex.	5.11, 5.11, 14.006	316.867	9008082	4.519
48 kHz	α-Ti	90.6	194: P63/mmc, hex.	2.958, 2.958, 4.750	36.002	9008517	4.416
	TiO	7.8	225: Fm-3m cubic (fcc)	4.194,	73.785	9008749	5.749
	Anatase TiO_2_	1.6	141: I41/amd, choice-1, tetragonal	3.782, 3.782, 9.514	136.124	9008213	3.897
20 kHz	Rutile Ti O_2_	23.0	136: P42/mnm tetragonal	4.656, 4.656, 3.031	65.725	9004144	4.036
	α-Ti	64.3	194: P63/mmc hex	2.931, 2.931, 4.705	35.020	9016190	4.539
	β-Ti	12.7	229: Im-3m cubic (bcc)	3.283	35.38	9011925	4.05
10 kHz	Ti_2_O	15	191: P6/mmm hex	5.068, 5.068, 2.882	64.123	9008976	4.340
	Ti_5_O_5_	14	12:112/m, unique-c, cell-1. monocl	5.856, 9.342, 4.143	216.177	9016272	4.906
	Rutile Ti O_2_	4	136: P42/mnm tetragonal	4.664, 4.664, 3.032	65.975	9004142	4.020
	α-Ti	40	194: P63/mmc hex	2.936, 2.936, 4.663	34.831	9016190	4.564
	α′-Ti	27	191: P6/mmm hex	4.493,4.493, 2.754	2.7546	9011600	4.950

**Table 4:** Volume loss, resistance to wear and coefficient of friction determined in the ball-on disk friction-wear test for titanium grade 1 before and after surface laser treatment at different pulse frequency.

Specimen	Δm [g]	V_L_ (m)	Wv (m)	V_L_ (op)	Wv (op)	Av.
		[mm^3^]	[mm^3^/Nm]	[mm^3^]	[mm^3^/Nm]	CoF
Ti grade 1	9 × 10^−4^	4.059	2.018 × 10^−2^	6.816	3.38 × 10^−2^	0.59
48 kHz	7 × 10^−4^	1.804	8.96 × 10^−3^	9.549	4.75 × 10^−2^	0.48
20 kHz	1 × 10^−4^	0.451	2.24 × 10^−3^	1.322	2.8 × 10^−3^	0.44
10 kHz	1 × 10^−4^	0.451	2.24 × 10^−3^	0.567	1.6 × 10^−3^	0.25

## Data Availability

The original contributions presented in this study are included in the article. Further inquiries can be directed to the corresponding author.
